# Association of G>A transition in exon-1 of alpha crystallin gene in age-related cataracts

**DOI:** 10.4103/0974-620X.60014

**Published:** 2010

**Authors:** S. G. Bhagyalaxmi, T. Padma, G. B. Reddy, K. R. K Reddy

**Affiliations:** Department of Genetics, Osmania University, Hyderabad, India; 1 National Institute of Nutrition, Hyderabad, India; 2 Sarojini Devi Eye Hospital and Institute of Ophthalmology, Hyderabad, India

**Keywords:** Age-related cataracts, alpha-crystallin, cortical cataract, mixed type, nuclear cataract, posterior subcapsular cataract, single strand conformation polymorphism, single nucleotide polymorphism

## Abstract

**Aim:**

To identify the presence of a known or novel mutation/SNP in Exon-1 (ex-1) of alpha crystallin *(CRYAA)* gene in different types of age-related cataract (ARC) patients.

**Materials and Methods:**

Single strand Conformation Polymorphism (SSCP) analysis was carried for the detection of single nucleotide polymorphism (SNP) in ex-1 of alpha crystallin (CRYAA) gene which was confirmed by sequencing.

**Results:**

The SSCP analysis of ex-1 of *CRYAA* gene revealed mobility shift in patients and controls, which was due to G>A transition at 6^th^ position in exon-1 of *CRYAA* gene. All the three genotypes, GG, AA and GA, were detected in patients and controls indicating that G>A substitution is polymorphic. The analysis showed significant risk for heterozygotes (GA) as compared to pooled frequencies of homozygotes (GG + AA), which was 1.81 times for all the types of cataracts in general and 2.5 times for Nuclear Cataract and twice for Cortical Cataract.

**Conclusion:**

The GA heterozygotes were at higher risk for developing NC and CC types of cataracts, where as the GG homozygotes for MT and AA homozygotes for PSC types were at risk. To our knowledge, an association of G>A transition found in ex-1 of *CRYAA* gene with ARC, with differential risk of genotypes for individual type of cataracts has not been reported previously.

## Introduction

Age-related eye diseases are the leading cause of visual impairment and blindness worldwide.[1,2] The most prevalent among age-related eye diseases is cataract formation.[3] Cataracts account for an estimated 16 million cases of blindness worldwide and half of all the cases originate from Africa and Asia.[4] Disruption in the cellular structure of the lens due to aging can affect lens transparency and the opacities can morphologically be distinguished into nuclear, cortical, posterior sub-capsular and mixed types.[5–7] Each of these cataracts has associated risk factors that include genetic and environmental factors. In most populations, the most commonly observed age-related cataract is nuclear (NC) followed by cortical (CC) and posterior subcapsular types (PSC).[8–13]

Cataracts are generally associated with breakdown of the lens micro-architecture. The short-range ordered packing of thecrystallins, which make up over 90% of soluble lens proteins, are important in maintaining lens transparency. The α-crystallin is a multimeric protein comprising of two gene products, αA and αB. *CRYAA* present on chromosome 21q encodes the αA-subunit, whereas αB is encoded by the *CRYAB* gene located on chromosome 11q.[14] The multimeric protein is important in maintaining the transparency of the lens, possibly by ensuring that the complexes formed by them and other proteins of the lens remain soluble.[14]

Previous epidemiological studies have established that cataract development is affected by various factors such as diabetes, female gender, sunlight or UV light exposure, myopia, nutritional deficiencies, trauma and steroid use.[3,4] Although, epidemiological studies reported the involvement of genetic factors in the pathogenesis of age-related cataracts,[13] the roles of specific genes causing susceptibility to the condition are not established and only a beginning is made with focus on unraveling underlying genetic basis. Over the past few years, attempts are being made for the identification of new loci for ARC. Identifying the genes and characterizing the proteins they encode will provide better comprehension of the molecular processes that are involved in the cataract development. There are now several reported mutations in congenital and hereditary cataracts involving genes that encode proteins with structural, metabolic, transport functions.[15–17] It is plausible that some of these genes may be involved in adult cataract with late expression of phenotype due to interaction with several noxious factors along with aging process.

## Materials and Methods

### Material

#### Patients

We conducted a case-control study of different types of ARC during the period 2003-2008 with the approval of the Ethical Committee of Department of Genetics, University College of Science, Osmania University, Hyderabad, India; 455 cases with different types of ARC (108-NC; 105-CC; 96-PSC; 146-MT) were screened for SNP variations in exon-1 of *CRYAA* gene. The patients studied were recruited from the Sarojini Devi Eye Hospital (SDEH) and Institute of Ophthalmology, Hyderabad, India after explaining to them the purpose of the study and obtaining informed consent.

#### Inclusion and exclusion criteria

Only patients with primary cataracts who underwent lens extraction were included in the present study. Diagnosis of different types of cataracts was done based on the Silt-lamp examination by the ophthalmologist following LOC-III classification.[18] Patients with secondary cataracts like acquired cataracts, caused due to trauma, toxins; and the complicated cataracts that occur due to inflammatory, and degenerative ocular diseases have been excluded from the study. In addition, patients with associated conditions like diabetes, hypertension, myopia, glaucoma, syndromes and patients under medications known to be associated with development of cataract (like steroids) were not considered.

#### Controls

A total of 144 randomly selected age and sex matched normal healthy individuals without history of cataract, diabetes, hypertension, and other ocular diseases were used as controls to compare with patient group.

#### Collection of data and blood samples

From all the patients and controls included in the study, information pertaining to sex, age, age at onset, duration of disease, type of cataract, any other associated conditions, information on habits, diet, detailed four generation family history, was collected using a specified proforma. The information collected was cross-matched with the accompanying relatives who were mostly first-degree relatives. 5-10 ml of venous blood samples were collected in EDTA vaccutainers from ARC patients and control subjects.

### Method

DNA was isolated using the salting out method.[19] PCR amplification (Applied Biosystem-9800 Fast Thermal Cycler) of exon-1 of *CRYAA* gene was performed with 50 ng of genomic DNA, in 100 µl PCR reaction mixture containing 10 µl 10XPCR buffer (100 mM Tris HCl, pH 8.8, 500 mM KCl, 15 mM MgCl2, 0.01% w/V gelatin); 200 µM each dATP, dGTP, dTTP, dCTP; 5.0 pmol of each primer and 2.5 U taq Polymerase using the following primer sequences forward primer - 5′-CTCCAGGTCCCCGTCCTACCA-3′ and reverse primer -5′-GCGAGGAGAGGCCAGCACCAC-3′,[20] which yielded 253 bp product with the PCR conditions- Initial denaturation 95°C, for five minutes, denaturation at 94°C for one minute, annealing at 52°C for one minute, and extension at 72°C for one minute, for 30 cycles, final extension at 72°C for five minutes. The PCR products were run on 0.8% agarose gel using ethidium bromide stain to test for the amplification and the bands were monitored under UV transilluminator. For Single strand Conformation Polymorphism (SSCP) analysis the PCR products were denatured and snap cooled immediately.

The denatured PCR products were analyzed by non-denaturing 12% polyacrylamide gel electrophoresis (PAGE) at 100 V for 16-18 hours at constant temperature using an Amarsham (Pharmacia Biotech SE600-15-1.5) electrophoresis unit. Silver staining was done to visualize the bands. When the mobility shift was detected, the exact nature of the SNP variation in the samples was confirmed by sequencing on an automated DNA sequencer (Applied Biosystem).

### Data analysis

The Ex-1 *CRYAA* polymorphisms studied in 455 cataract patients with different types of ARCs and 144 controls, were analyzed by Chi-square test of significance and Odds Ratio (OR) with 95% confidence Interval (CI) to estimate the risk of specific genotypes for the development of different types of cataracts. In order to assess the risk of the genotypes for developing age-related cataracts (ARCs) in association with the confounding factors, the data was subjected to regression analysis considering ARCs as dependable variable and other confounding factors as independent variables applying Fisher′s correction to reduce the sample bias.

## Results

The base line characteristic features of cases with ARC (ARC) studied showed, high predilection of females (54.7%) as compared to males (45.3%) in almost all the types of cataracts [Table 1]. The mean ages-at-onset of cataracts were 59.8 ± 0.92 in (NC); 57.02 ± 01.1 in (CC); 54.5 ± 1.22 in (PSC) and 57.4 ± 0.80 in (MT) and 51.3 ± 10.7 in controls (mean ± Stdev). The incidence of presenile cases (age-at-onset <50 years) was high in PSC (34.0%) and CC (23.0%) as compared to NC (6.5%). Only male subjects from cases (455) and control (144) were reported to be smokers. The frequency of smokers was high in patients with NC (53.0%) compared to CC (47.0%), PSC (38.0%) and controls (37.0%). Table 1 also provides the other information such as the frequency of alcohol consumption, dietary habits in patients and control subjects.

**Table 1:** Baseline characteristic features found in different types of age-related cataracts cases and control subjects studied

*Age-related cataract cases*											*Controls (144)*	
	*NC (108)*		*CC (105)*		*PSC (96)*		*MT (146)*		*Total (455)*		*N*	*%*
	*N*	*%*	*N*	*%*	*N*	*%*	*N*	*%*	*N*	*%*		
Males	45	42.0	58	55.0	42	44.0	61	42.0	206	45.3	85	59.0
Females	63	58.0	47	45.0	54	56.0	85	58.0	249	54.7	59	40.9
Presenile	07	6.5	24	23.0	33	34.0	33	23.0	97	21.0	-	-
Senile	101	93.5	81	77.0	63	66.0	113	77.0	358	79.0	-	-
Smoker	24	53.0	27	47.0	16	38.0	26	43.0	93	45.0	31	37.0
Non-smoker	21	47.0	31	53.0	26	62.0	35	57.0	113	55.0	54	63.0
Alcoholics	25	23.0	19	18.0	20	21.0	36	25.0	100	22.0	40	28.0
Non-alc	83	77.0	86	82.0	76	79.0	110	75.0	355	78.0	104	72.0
Veg	10	9.3	19	18.1	05	5.2	11	7.5	45	9.9	11	7.6
Non-veg	98	90.7	86	81.9	91	94.8	135	92.5	410	90.1	133	92.4

For smokers data, only male subjects of cases and controls were reported to be smokers; NC - Nuclear cataracts; CC - Cortical cataracts; PSC - Posterior sub- capsular cataracts; MT - Mixed type cataracts

**Figure 1:**
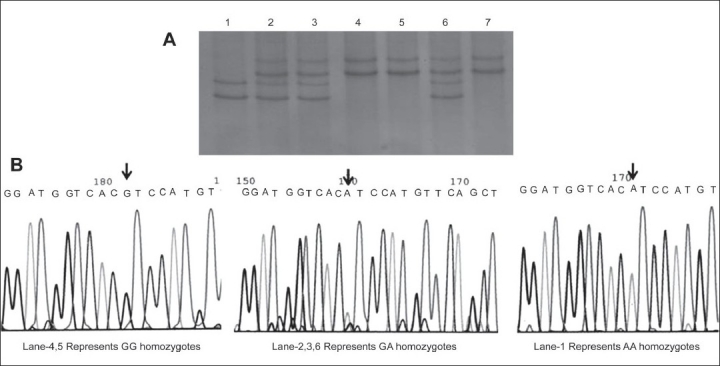
Panel-A and Panel-B. Panel A - SSCP analysis revealing mobility shift showing three different patterns of bands for G > A polymorphisms. Lane-4 and 5 represents GG homozygotes, lane-2, 3, 6 represents GA heterozygotes, lane-1 represents AA homozygote. Panel-B - Electrophorograms showing Homozygous GG, Heterozygote GA and Homozygous AA polymorphisms in Exon-1 of alpha crystallin gene. The arrow points to the variant nucleotide

The SSCP analysis revealed mobility shift showing three different patterns of bands in patients and as well as in controls. Sequence analysis identified a G>A transition at 6^th^ position in exon-1 of *CRYAA* gene (counting the A of the start codon as number-1) that leads to the synonymous codon (GAG to GAA) coding for the same amino acid - Aspartic acid (D). Since this substitution, G>A was found in both patients and control group, the change was considered as polymorphic rather than a causative mutation. Based on this substitution (G>A) the individuals were genotyped as homozygote GG (with two slow moving cathodic bands), homozygote AA (with two fast moving anodic bands) and heterozygote GA (with four bands corresponding to GG and AA genotypes) [Figure 1: Panel-A and Panel-B].

From Table 2 it may be observed that there is a high frequency of GG genotypes in different types of ARC cases and controls (Total-67.5%; NC-58.0%; CC-68.0%; PSC-72.0%; MT-70.5%; controls-81.0%) followed by GA (Total-27.0%; NC-33.0%; CC-29.0%; PSC-20.0%; MT-24.6%; controls-17.0%) and AA genotypes (Total-6.2%; NC 8.3%; CC-3.8%; PSC-8.3%; MT-4.7%; controls-2.0%).

**Table 2:** Distribution of G>A Polymorphism in exon-1 of CRYAA gene in different types of age-related cataract cases and control studied

	*GG*		*GA*		*AA*		*Total*		*Allele frequency*				*Cases Vs control*		*H.W. equilibrium*	
	*N*	*%*	*N*	*%*	*N*	*%*	*N*	*%*	*G-allele freq.*		*A-allele freq.*		*χ^2^*	*P-value*	*X^2^*	*P value*
									*N*	*%*	*N*	*%*				
NC	63	58.0	6	33.0	09	8.3	108	24.0	81.0	0.75	27.0	0.25	16.8	0.002	1.33	≥0.05
CC	71	68.0	30	29.0	04	3.8	105	23.0	86.0	0.82	19.0	0.18	6.1	0.40	1.13	≥0.05
PSC	69	72.0	19	20.0	08	8.3	96	21.0	78.5	0.82	17.5	0.18	5.87	0.05	10.8^*^	≤0.05
MT	103	70.5	36	24.6	07	4.7	146	32.0	121.0	0.83	25.0	0.17	4.87	0.08	2.5	≥0.05
Total	306	67.5	121	27.0	28	6.2	455		366.5	0.81	88.5	0.19	10.9	0.004	10.4^*^	≤0.05
Controls	117	81.0	24	17.0	03	2.00	144		129	0.89	15	0.10	-	-	1.64	≥0.05

NC - Nuclear cataracts; CC - Cortical cataracts; PSC - Posterior sub-capsular cataracts; MT - Mixed type cataracts

As compared to controls the patients showed a high frequency of GA heterozygotes (patients-27.0%; controls-17.0%) and specifically in patients with NC (33.0%) and CC (29.0%) types of cataract. The frequency of AA genotypes was also higher in patient group (6.2%) as compared to controls (2.0%) and specifically in patients with NC (8.3%) and PSC (8.3%). In general, the distribution of GG, GA and AA genotypes in patients and different types of cataracts deviated significantly when compared to controls (χ^2^ -10.9 *P* ≤ 0.004) except for the MT type of cataract (NC-χ^2^ -16.8; *P* ≤ 0.002; CC-χ^2^ -6.1; *P* ≤ 0.04; PSC-χ^2^ -5.87; *P* ≤ 0.05; Table 2. This finding suggests that the heterozygosity with G>A transition may be associated with ARC.

Testing the data for HW equilibrium, significant deviation for patient group was found in general (10.4; *P* ≤ 0.05) and specifically for PSC (10.8; *P* ≤ 0.05) and not for controls [Table 2]. This result also indicates that the G>A transition may be associated with ARC.

When the distribution of GG, GA, AA types were tested for their association with reference to different stratified groups, significant difference was found when male cataract patients were compared to male controls (χ^2^ -14.4;*P* ≤ 0.0007) with high frequency of heterozygotes. This could be due to the increased frequency of GA genotypes found in cataract patients (27.0%) as compared to controls (17.0%) [Table 2] . The distribution did not vary significantly when other stratified groups were compared between patients and controls and within patients and within controls.

Odds ratios were performed by pooling the frequencies of genotypes in different combinations like, GG Vs GA + AA; GA Vs GG + AA; AA Vs GA + GG, to evaluate the extent of risk for specific genotype for developing cataracts in relation to other combinations [Table 3] . The analysis showed significant risk for heterozygotes (GA) as compared to pooled frequencies of homozygotes (GG + AA), which was 1.81 times for all the types of cataracts in general and 2.5 times for NC and twice for CC.

**Table 3:** Odds ratios for different genotypes of G>A substitution in different types of age-related cataracts as compared to controls

	*GG Vs (GA + AA)*				*GA Vs (GG + AA)*				*AA Vs (GG + GA)*				*G/A*			
	*OR*	*95% CI*	*x^2^*	*P value*	*OR*	*95% CI*	*x^2^*	*P value*	*OR*	*95% CI*	*x^2^*	*P-value*	*OR*	*95% CI*	*x^2^*	*P value*
NC	0.32	0.18-0.56	16.8	0.00006	2.5	1.38-4.52	9.45	0.005	4.27	1.12-16.2	4.02	0.04	0.34	0.17-0.69	9.45	0.002
CC	0.44	0.24-0.78	6.1	0.01	2	1.08-3.67	5.06	0.02	1.86	0.40-8.49	0.18	0.67	0.45	0.22-0.92	3.03	0.08
PSC	0.58	0.32-1.08	2.9	0.08	1.23	0.63-2.40	0.38	0.53	4.27	1.10-16.5	3.81	0.05	0.52	0.24-1.09	3	0.08
MT	4.75	1.76-12.8	6.17	0.01	1.63	0.91-2.91	2.82	0.09	2.36	0.59-9.33	0.89	0.34	0.56	0.28-1.11	2.7	0.09

NC - Nuclear cataracts; CC - Cortical cataracts; PSC - Posterior sub-capsular cataracts; MT - Mixed type cataracts

When odds ratios for other combinations were considered, significantly low risk was observed for GG genotypes in relation to others in all the types of cataracts except for MT type (NC- OR-0.32; 95% CI 0.18-0.56; X^2^ -16.8, *P* ≤ 0.00006; CC-OR-0.44; 95% CI 0.24-0.78; X^2^ -6.1, *P* ≤ 0.01; PSC- OR-0.58; 95% CI-0.32-1.08; MT-4.75; 95% CI 1.76-12.8; X^2^- 6.17, *P* ≤ 0.01; Table 3). When the AA genotype was compared with GG + GA genotypes, it revealed high risk for patients with NC and PSC types of cataracts (NC -OR-4.27; 95% CI 1.12-16.2; X^2^- 4.02, *P* ≤ 0.04; PSC- OR-4.27; 95% CI 1.10-16.5; X^2^- 3.81, *P* ≤ 0.05; Table 3).

In order to assess the risk of the genotypes for developing age-related cataracts (ARCs) in association with the confounding factors, the data was subjected to regression analysis considering ARCs as dependable variable and other confounding factors as independent variables for adjusting after sample bias. The results indicated GA heterozygosity and female sex as significant predictor for ARCs (regression coefficient value-0.6602 with SE-0.221; Chi-square value- 8.88; *P*-value - 0.002).

## Discussion

Age-related cataracts (ARC) are one of the leading causes of visual impairment and blindness among the elderly worldwide.[21] Genetic predisposition caused by several genes interacting with different epidemiological factors is considered as determinants of ARC making the condition multifactorial. Even when the risk factors can be controlled, genetic predisposition could influence the onset of the condition to a greater extent.

Although epidemiological research has focused mostly on the role of environmental risk factors in ARC, recent studies have provided evidence for the contribution of genetic factors in the pathogenesis of ARC.[22,23] A study by Iyenger,[7] *et* al. identified several loci through the genome scan on age-related cortical cataracts; although, these loci did not coincide with the map coordinates of known crystalline genes. The study also revealed that weaker loci are critical for age-related cortical cataract or act as modifiers, and suggested that the pathways and genes that are less crucial to the development of the lens fibers may play a role in ARC.

It is well recognized that α-crystallins function like a chaperone and play a decisive role in the maintenance of the eye lens transparency.[24,25] Conversely, it is well established that certain point mutations in αA- and αB-crystallin genes are linked with non-syndromic, hereditary human cataracts.[24,25] So far, mutations in ex-1 of *CRYAA* gene have been reported in congenital cataracts like the W9X-mutation,[20] and the R49C mutation.[26] But there are no reports on CRYAA gene mutations in age-related cataracts.

We report a SNP (G>A transition) in Ex-1 of *CRYAA* gene, which may be presumed to be responsible for the molecular changes leading to cataract formation in aged people. The GA heterozygotes were strongly associated with the development of NC and CC types of cataracts, where as the GG homozygotes were strongly associated for MT type of cataract and AA homozygotes were at risk for PSC types of cataracts. To our knowledge, this is the first report on the association of G>A transition at 6^th^ position in exon-1 of *CRYAA* gene with ARC, where the genotypes showed differential risk for individual type of cataracts. This finding is interesting and needs further studies to confirm its presence and its implications as a marker for studying ARCs.

Analysis of the sequence of the human genome has shown that the extent of genetic variation in the human population is far greater than had been estimated,[27] and the most common sequence variation is the single-nucleotide polymorphism (SNP). SNP in definition is a minor allele frequency of >1% that is the major determinant of variations in disease susceptibility, response to medication, and toxicity.[28] As SNPs occur at a frequency of 1 per 100 to 1000 bp,[27] several strategies have been advocated to systematically or rationally reduce the number of SNPs that need to be studied. The synonymous SNPs (which do not alter amino acid residues) are based on the assumption that they are "silent" and do not affect protein expression or function.

Polymorphisms in the human genome contribute to wide variations in how individuals respond to medications, either by changing the pharmacokinetics of drugs or by altering the cellular response to therapeutic agents. The goal of the emerging discipline of Pharmacogenomics is to personalize therapy based on an individual′s genotype. Due to the relatively large frequency of single-nucleotide polymorphisms (SNP) in the human genome, synonymous SNPs are often disregarded in many pharmacogenomic studies based on the assumption that these are silent.

It has been recently shown,[29] that synonymous SNPs in ABCB1 (P-glycoprotein), implicated both in determining drug pharmacokinetics and multidrug resistance in human cancer cells, can affect protein conformation and function. The importance of polymorphisms in drug metabolizing enzymes and transporters in anticancer therapy suggest that synonymous polymorphisms may play a more significant role than is currently assumed.

Likewise, these silent SNP may be involved in adult cataract formation with late expression of phenotype, due to interaction with several noxious factors along with aging process. Understanding the role of this silent SNP for its role in modifying the conformation of Alpha crystalline protein and its function would help determine the drug pharmacokinetics which could be useful in understanding the alterations of lens crystallins leading to cataract formation. This can probably provide the evidence for the genetic factors in the pathogenesis of ARCs and response of individual genotypes to medications in terms of toxicity and efficacy.
